# Primary Breast Lymphoma in Mexico: A 15-Year Retrospective Case Series From a Tertiary Center Suggesting a Distinctly Early Age at Presentation

**DOI:** 10.7759/cureus.104497

**Published:** 2026-03-01

**Authors:** Javier A Teco-Cortes, Juan J Navarrete-Pérez, Oscar E Sánchez-Castro, Omar de los Santos-Farrera

**Affiliations:** 1 Department of Pathology, Instituto Nacional de Ciencias Médicas y Nutrición "Salvador Zubirán", Mexico City, MEX; 2 Department of Pathology, Hospital General de México "Dr. Eduardo Liceaga", Mexico City, MEX; 3 Department of Pathology, Hospital General La Villa, Mexico City, MEX

**Keywords:** breast, breast lymphoma, diffuse large b-cell lymphoma, mexico, primary breast lymphoma

## Abstract

Background: Primary breast lymphoma (PBL) is a rare extranodal lymphoma involving breast tissue and most commonly affects women in the sixth to seventh decades of life. We describe a clinicopathological series of PBL cases diagnosed at a tertiary referral center in Mexico. Histologically, most cases correspond to B-cell non-Hodgkin lymphomas, particularly diffuse large B-cell lymphoma (DLBCL). Due to its rarity, available evidence is largely derived from small case series and retrospective analyses, highlighting the need for additional reports from diverse populations.

Methods: A retrospective, observational, descriptive study was conducted at a tertiary referral center in Mexico. All cases diagnosed as PBL between 2005 and 2020 were retrieved from the institutional pathology database. Inclusion criteria consisted of a histopathological diagnosis of primary lymphoma involving the breast with availability of complete histopathological material, including paraffin blocks and immunohistochemical studies. Descriptive statistics were used. Continuous variables are expressed as mean ± standard deviation, and categorical variables as absolute frequencies and percentages. Due to the small sample size, no inferential statistical analysis was performed.

Results: Six cases met the diagnostic criteria for PBL (N = 6), and all patients were women. The mean age at diagnosis was 38.2 ± 19 years (range, 19-66). DLBCL was the most frequent subtype (4/6, 66.7%), followed by classical Hodgkin lymphoma (1/6, 16.7%) and B-lymphoblastic lymphoma (1/6, 16.7%). Left breast involvement was observed in three cases, right-sided disease in one, bilateral presentation in one, and one case lacked specified laterality.

Conclusions: In this single-center Mexican series, patients were diagnosed at a younger age than typically reported in the international literature. DLBCL was the predominant subtype. These findings suggest the possibility of population-specific epidemiological patterns and underscore the need for larger regional studies to clarify potential differences in disease presentation.

## Introduction

Breast lymphomas are rare hematolymphoid malignancies, partly due to the limited native lymphoid tissue within the breast and the absence of clearly established risk factors for this site. They are classified as primary or secondary according to the criteria proposed by Wiseman and Liao in 1972 [1]. According to these criteria, a diagnosis of primary breast lymphoma (PBL) requires confinement of the neoplasm to the breast as the principal site of disease, with or without ipsilateral regional lymph node involvement, no prior history of lymphoma or disseminated disease at diagnosis, and histopathological confirmation of lymphoma within breast tissue.

PBL accounts for 0.04% to 0.5% of all malignant breast neoplasms and approximately 2% of extranodal lymphomas, with a reported increasing incidence in recent years. Although it may occur at any age, it predominantly affects middle-aged or older women, typically between 60 and 65 years. Clinical presentation may include a solitary mass, multiple masses, or diffuse involvement, with up to 11% of cases presenting bilaterally. Clinical and imaging findings are usually nonspecific, often mimicking breast carcinoma [2,3].

Most cases correspond to diffuse large B-cell lymphoma (DLBCL) (40%-73%), followed by extranodal marginal zone lymphoma of mucosa-associated lymphoid tissue (MALT lymphoma) (9%-25%) and follicular lymphoma (FL) (13%-19%). Less frequently, Burkitt lymphoma and breast implant-associated anaplastic large cell lymphoma have been reported, although virtually any lymphoma subtype defined by the WHO classification may occur [2,4,5].

In Mexico, epidemiological data on PBL are limited due to its rarity, and knowledge is largely restricted to isolated case reports. The objective of this study is to present a small clinicopathological series that may serve as an initial step toward larger investigations in Mexico and other regions.

## Materials and methods

A retrospective, observational, descriptive study was conducted at the Department of Pathology, Hospital General de México “Dr. Eduardo Liceaga,” Mexico City, Mexico. Cases diagnosed as PBL between 2005 and 2020 were identified from the institutional pathology database.

Given the rarity of PBL, all available cases diagnosed during the study period were included. No formal sample size calculation was performed due to the descriptive, retrospective nature of the study. Consecutive non-probability sampling was used to include all eligible cases.

Clinical records and imaging studies were requested but were unavailable due to institutional policies regarding long-term storage. Histopathological reports and archived slides, preserved in the pathology department, were reviewed. Diagnoses were confirmed or reclassified when appropriate. All cases were reviewed by certified pathologists, and histological classification was established according to the 5th edition of the World Health Organization (WHO) Classification of Hematolymphoid Tumors. 

Inclusion criteria included histopathological diagnosis of PBL, diagnosis between 2005 and 2020, and availability of paraffin blocks and histologic slides. Exclusion criteria included secondary breast lymphoma, incomplete histopathological data, and if the histopathological review did not confirm PBL.

Recorded variables included age, sex, laterality, and histopathological subtype. Descriptive statistics were used for analysis, with continuous variables expressed as mean ± standard deviation and categorical variables as frequencies and percentages.

The study was conducted in accordance with the Declaration of Helsinki and approved by the institutional ethics committee. Patient confidentiality was maintained throughout the study.

## Results

Seven cases were initially identified. Following histopathological review, one case was excluded because it corresponded to a cutaneous pseudolymphoma involving the breast.

The remaining six cases were confirmed as PBLs. All patients were women (100%), with ages ranging from 19 to 66 years (mean ± SD: 38.2 ± 19 years).

Laterality was specified in five cases: three involved the left breast (50%), one the right breast (16.7%), and one presented with synchronous bilateral involvement (16.7%). Laterality was not specified in one case (16.7%).

**Figure 1:**
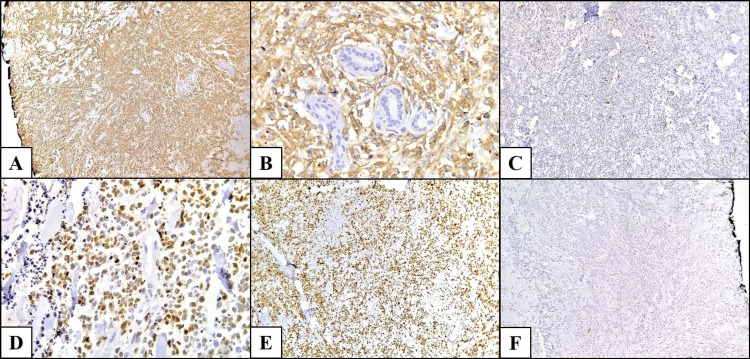
Diffuse large B-cell lymphoma involving the breast (A) Diffuse solid proliferation of large atypical lymphoid cells showing strong CD20 positivity (immunoperoxidase stain, 40×). (B) Higher magnification demonstrating CD20-positive neoplastic cells surrounding residual mammary ducts (immunoperoxidase stain, 400×). (C) Neoplastic cells are negative for CD5 (immunoperoxidase stain, 40×). (D) Diffuse nuclear positivity for MUM1 in tumor cells (immunoperoxidase stain, 100×). (E) Ki-67 proliferation index of 80% in neoplastic cells (immunoperoxidase stain, 40×). (F) Tumor cells are negative for cytokeratin AE1/AE3 (immunoperoxidase stain, 40×). Note: These are original histopathological images from Case 6 in this study.

**Figure 2:**
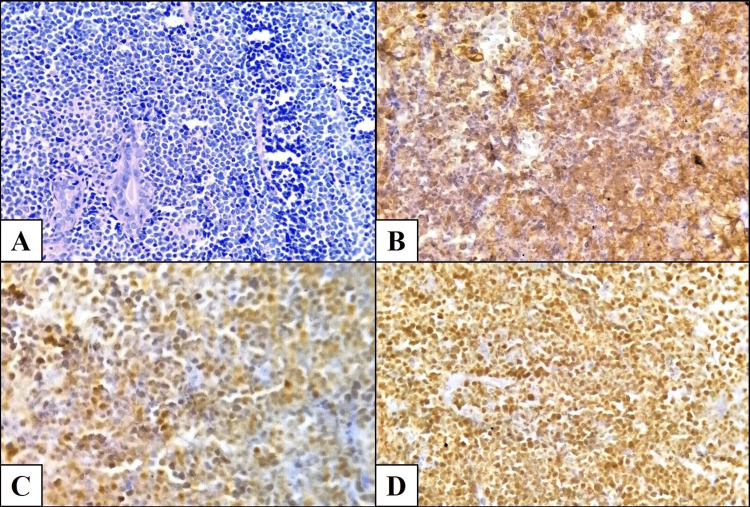
B-lymphoblastic lymphoma of the breast (A) Diffuse solid proliferation of small- to medium-sized non-cohesive lymphoid cells with scant cytoplasm and basophilic nuclei, surrounding residual mammary ducts (hematoxylin and eosin stain, 40×). (B) Tumor cells showing positivity for CD79a (immunoperoxidase stain, 100×). (C) Nuclear positivity for TdT in neoplastic cells (immunoperoxidase stain, 100×). (D) Tumor cells positive for PAX5 (immunoperoxidase stain, 100×). Note: These are original histopathological images from Case 4 in this study.

Regarding histopathological subtypes, four cases were DLBCL (66.7%) (Figure 1), one was B-lymphoblastic lymphoma (16.7%) (Figure 2), and one was classical Hodgkin lymphoma, nodular sclerosis subtype (16.7%) (Table 1).

**Table 1:** Clinicopathological features of primary breast lymphoma in a Mexican tertiary referral center (2005-2020) (N = 6) Data are presented as absolute numbers. Categorical variables are expressed as frequency (percentage).

Case	Sex	Age (years)	Laterality	Histopathological diagnosis	Positive IHC markers
1	Female	20	Right	Diffuse large B-cell lymphoma	CD20, PAX5, CD10, BCL6, Ki-67 (30%)
2	Female	34	Left	Classical Hodgkin lymphoma, nodular sclerosis subtype	PAX5, CD30, CD15, MUM1
3	Female	66	Not specified	Diffuse large B-cell lymphoma	CD20, PAX5, BCL6, Ki-67 (40%)
4	Female	19	Bilateral	B-lymphoblastic lymphoma	CD19, CD79a, Tdt, CD10, PAX5
5	Female	59	Left	Diffuse large B-cell lymphoma	CD20, PAX5, CD10, MUM1, Ki-67 (50%)
6	Female	31	Left	Diffuse large B-cell lymphoma	CD20, CD10, MUM1, Ki-67 (80%)

## Discussion

PBL is a rare neoplasm with limited epidemiological and prognostic data, particularly in Latin America. Consistent with prior reports, all cases in our series occurred in women. International literature reports an average age at diagnosis of 60-65 years [2,6], which contrasts sharply with the mean age of 38.2 years in our series. This notable difference underscores the need for larger clinicopathological studies in our population to better understand factors associated with earlier presentation.

Regarding laterality, secondary breast lymphoma is more frequently reported in the right breast, whereas PBL appears more commonly in the left breast [3], consistent with our findings. Bilateral involvement has been reported in up to 11% of cases [2,7,8], similar to our series (16.7%), though this represented a single patient.

DLBCL is the most frequent subtype of PBL, reported in up to 73% of cases, comparable to the 66.7% observed in our series. Unlike expectations, we did not identify follicular or MALT lymphomas, but instead observed less common subtypes. DLBCL of the breast typically presents in the seventh decade, with no clear laterality predilection, and up to 5% of cases are bilateral. Its pathogenesis at this site remains unclear. Gene expression profiling classifies DLBCL into germinal center B-cell (GCB), activated B-cell (ABC), and unclassifiable subtypes. Reported five-year overall survival ranges from 70% to 80%, with disease-free survival between 60% and 65% [2,9,10]. In our series, the mean age for DLBCL cases was 44 ± 19 years, markedly younger than reported internationally.

Primary Hodgkin lymphoma of the breast is exceedingly rare, with only isolated case reports. The nodular sclerosis variant is most frequently described, typically affecting postmenopausal women and sometimes associated with Epstein-Barr virus. Prognostic data remain limited [11,12]. Our case corresponded to the nodular sclerosis subtype in a 34-year-old woman, consistent with previously reported younger cases [13].

Primary B-lymphoblastic lymphoma of the breast is even rarer, with only four prior cases reported. Two occurred in young women aged 14 and 18 years; one involved the right breast, while laterality was unspecified in the other [14,15]. The remaining two cases were part of a series by Pérez et al., representing 3.63% of lymphomas in their cohort, though no additional clinical details were provided [10]. In our series, this subtype occurred in a 19-year-old patient with bilateral involvement, representing the fifth reported case to date.

This series is limited by its small size and the absence of complete clinical data due to institutional archival policies. Nevertheless, it provides relevant observations, including a markedly younger age at presentation and the identification of uncommon histological subtypes. Although data from Latin America remain scarce, previously published reports generally describe age distributions similar to international cohorts [16,17], suggesting potential regional variability that warrants further investigation.

From a diagnostic standpoint, breast lymphoma represents both a clinical and pathological challenge. The differential diagnosis includes reactive or infectious conditions, such as mastitis, medullary carcinoma with prominent lymphoid infiltrate, neuroendocrine carcinoma, and lobular carcinoma with discohesive cells that may mimic lymphoma [4]. Signet-ring morphology may also be observed in lymphomas [18]. Therefore, a thorough histopathological evaluation combined with immunohistochemistry is essential. Systemic involvement must always be excluded before establishing the diagnosis of PBL.

Study limitations

This study has several limitations. First, the small sample size (N = 6) limits the generalizability of our findings. Second, due to institutional archival policies, complete clinical and imaging records were unavailable for all patients. Finally, as a retrospective single-center study, selection bias cannot be excluded. Despite these limitations, the series provides valuable insight into PBL epidemiology and pathology in a Latin American population and highlights the need for larger regional studies.

## Conclusions

PBLs are rare neoplasms that must be recognized to avoid misdiagnosis as inflammatory conditions or breast carcinomas. Our series demonstrates a markedly younger age at presentation and the presence of uncommon histological subtypes compared with international data. These findings suggest potential population-specific epidemiological differences and underscore the need for larger studies to better define risk factors, biological behavior, and prognosis, as well as to emphasize the importance of early and accurate diagnosis.
